# Patients with infective endocarditis and history of injection drug use in a Swedish referral hospital during 10 years

**DOI:** 10.1186/s12879-021-05914-1

**Published:** 2021-03-02

**Authors:** Anna Damlin, Katarina Westling

**Affiliations:** 1 Department of Molecular Medicine and Surgery, Division of Clinical Physiology, Karolinska Institutet, SE-171 76 Stockholm, Sweden; 2 Department of Clinical Physiology, Karolinska University Hospital, A8:01, Eugeniavägen 3, SE-171 76 Stockholm, Sweden; 3 Department of Medicine Huddinge, Division of Infectious Diseases and Dermatology, Karolinska Institutet, SE-141 86 Stockholm, Sweden; 4 Department of Infectious Diseases, Karolinska University Hospital Huddinge, SE-141 86 Stockholm, Sweden

**Keywords:** Infective endocarditis, Injection drug use, Echocardiography

## Abstract

**Background:**

Patients with injection drug use (IDU) have increased risk of developing infective endocarditis (IE). Previous studies have reported recurrent IE, increased duration of hospital stay, poor adherence and compliance as well as higher mortality and worse outcomes after surgery in the IDU-IE patient group. Further studies are needed to provide a basis for optimized care and prevention of readmissions in this population. This study aims to describe the clinical characteristics and outcomes among patients with IDU-IE.

**Methods:**

Data of adults with IDU-IE and non-IDU-IE, treated between 2008 and 2017 at the Karolinska University Hospital in Stockholm were obtained from the Swedish National Registry of Infective Endocarditis. Clinical characteristics, microbiological results, treatment durations, results from echocardiography and in-hospital mortality were compared between the groups.

**Results:**

Of the total 522 patients, 165 (32%) had IDU-IE. Patients with IDU-IE were younger than the patients with non-IDU-IE (mean age IDU-IE: 41.6 years, SD 11.9 years; non-IDU-IE: 64.3 years, SD 16.4 years; *P* <  0.01). No difference in distribution of gender was observed, 33% were females in both the IDU-IE and the non-IDU-IE group. History of previous IE (IDU-IE: *n* = 49, 30%; non-IDU-IE: *n* = 34, 10%; *P* <  0.01) and vascular phenomena (IDU-IE: *n* = 101, 61%; non-IDU-IE: *n* = 120, 34%; *P* <  0.01) were more common among patients with IDU-IE while prosthetic heart valves (IDU-IE: *n* = 12, 7%; non-IDU-IE: *n* = 83, 23%; *P* <  0.01) and known valvular disease (IDU-IE: *n* = 3, 2%; non-IDU-IE: *n* = 78, 22%; *P* <  0.01) were more common among patients with non-IDU-IE. Aetiology of *Staphylococcus aureus* (IDU-IE: *n* = 123, 75%; non-IDU-IE: *n* = 118, 33%; *P* <  0.01) as well as tricuspid (IDU-IE: *n* = 91, 55%; non-IDU-IE: *n* = 23, 6%; *P* <  0.01) or pulmonary valve vegetations (IDU-IE: *n* = 7, 4%; non-IDU-IE: n = 2, 1%; *P* <  0.01) were more common in the IDU-IE group. The overall incidence of IDU-IE decreased during the study period, while the incidence of definite IE increased (*P* <  0.01).

**Conclusions:**

This study presents that patients with IDU-IE were younger, less frequently treated with surgery and had higher prevalence of vascular phenomena and history of previous IE, aspects that are important for improved management of this population.

## Background

Infective endocarditis (IE) is one of the infectious diseases with the highest morbidity and mortality [1–4]. In several countries, such as the United States, the incidences of IE have been reported as increasing [1–4]. One factor contributing to the increase of IE is the increasing incidence of injection drug use (IDU) [1–3]. People with IDU are at 100-fold increased risk of developing IE compared with the general population [1].

There are several mechanisms that can explain the increased risk of IE among patients with IDU: direct injury from injected substances, poor hygiene during injection, contaminated injection equipment, and physiological factors associated with IDU such as vasospasm and cardiac damage have also been described [2, 3, 5, 6]. IDU-related IE (IDU-IE) more often present with right-sided vegetations, rather than left-sided. The exposure from injected substances causing endothelial damage has been reported as the mechanism behind the right-sided manifestations, which most often present as tricuspid valve vegetations [3, 6, 7].

Previous studies have reported re-infections and relapses of IE, increased duration of hospital stay and poor adherence and compliance in the IDU-IE patient group [8–11]. Compared with patients with IE not related to IDU, cardiac surgery has been associated with higher mortality, worse risk-adjusted outcomes after surgery, and higher reoperation during the first 6 months after surgery in patients with IDU-IE [9, 12]. The risks of continuing addiction and IDU in this patient group adds further complexity that needs to be taken into account in the management of patients with IDU-IE, however often inadequately addressed [13]. Further studies are needed to better understand the characteristics and outcomes among the patients with IDU-IE.

This study aimed to present the clinical characteristics and outcomes among the patients with IDU and definite IE admitted to a university hospital in Stockholm between 2008 and 2017. The hospital has a special ward for drug addicts with infectious diseases, which contributes to a high prevalence of IDU among the patients with IE, which was presented in a previous study [14]. This enables good comparability between patients with IDU-IE and patients with IE without IDU, referred to as non-IDU-IE.

## Methods

### Study setting

This study is based on data from patients with IDU and IE, admitted to the Karolinska University Hospital in Stockholm, Sweden. The hospital has a special ward for drug addicts with infectious diseases, with an uptake area covering Stockholm County Council with approximately 1.9 million adult (18 years and above) inhabitants. The ward is operated by infectious diseases consultants and addiction psychiatrists. According to guidelines, most of the patients with IE at the hospital are discussed within an endocarditis team, comprising specialists in infectious diseases, cardiology, clinical physiology and thoracic surgery [15]. The majority of the echocardiographic examinations were conducted at the Department of Clinical Physiology, a unit specialized in cardiovascular imaging.

### Study design

This study is registry-based on data from the Swedish National Registry of Infective Endocarditis (SRIE). The SRIE is a national registry established in 1995, covering data from patients treated for possible or definite IE (ICD codes I33.0, I33.9, I38.9, and I39.8) in all Swedish departments for infectious diseases, with an estimated coverage of 70–80% [16]. In this study, data of patients with definite IE (patients with possible IE were excluded), treated at the Karolinska University Hospital in Stockholm, between 1 January 2008 and 31 December 2017 were obtained from the SRIE. Patients aged 18 years and above with registered history of IDU were selected for further analysis and compared with patients with non-IDU-IE. Age, gender, date of admission, clinical characteristics, congenital heart disease, co-morbidities, results from blood cultures and cultures or polymerase chain reaction (PCR) amplification from material sampled during cardiac surgery, results from echocardiographic examinations (transthoracic echocardiography: TTE and transesophageal echocardiography: TEE), antibiotic treatment durations, and in-hospital mortality were obtained from the SRIE. Coagulase-negative staphylococci (CoNS) were grouped including *Staphylococcus lugdunensis* and *Staphylococcus epidermidis*. The review of data from the registry was performed by one person (AD) and discussed with a specialist in infectious diseases (KW). All methods were performed in accordance with the relevant guidelines and regulations [15].

### Statistical methods

Continuous variables were described as means and standard deviations (SDs) or median and 25th and 75th percentiles and categorical variables as proportions (percentages). Mann-Whitney’s test was used for comparisons of means. For comparisons of medians, quantile regression was used. For comparisons of categorical variables, chi-squared test was conducted for values ≥5 and the two-sided Fisher’s exact test for values < 5. Logistic regression models were used to analyse in-hospital mortality in different patient groups. Variations over time were analysed using linear regression. In all analyses, *P*-values < 0.05 (two-tailed) were considered statistically significant. Analyses were performed using STATA software (version 15.1 Stata Corp., College Station, Texas, USA).

## Results

**Fig. 1:**
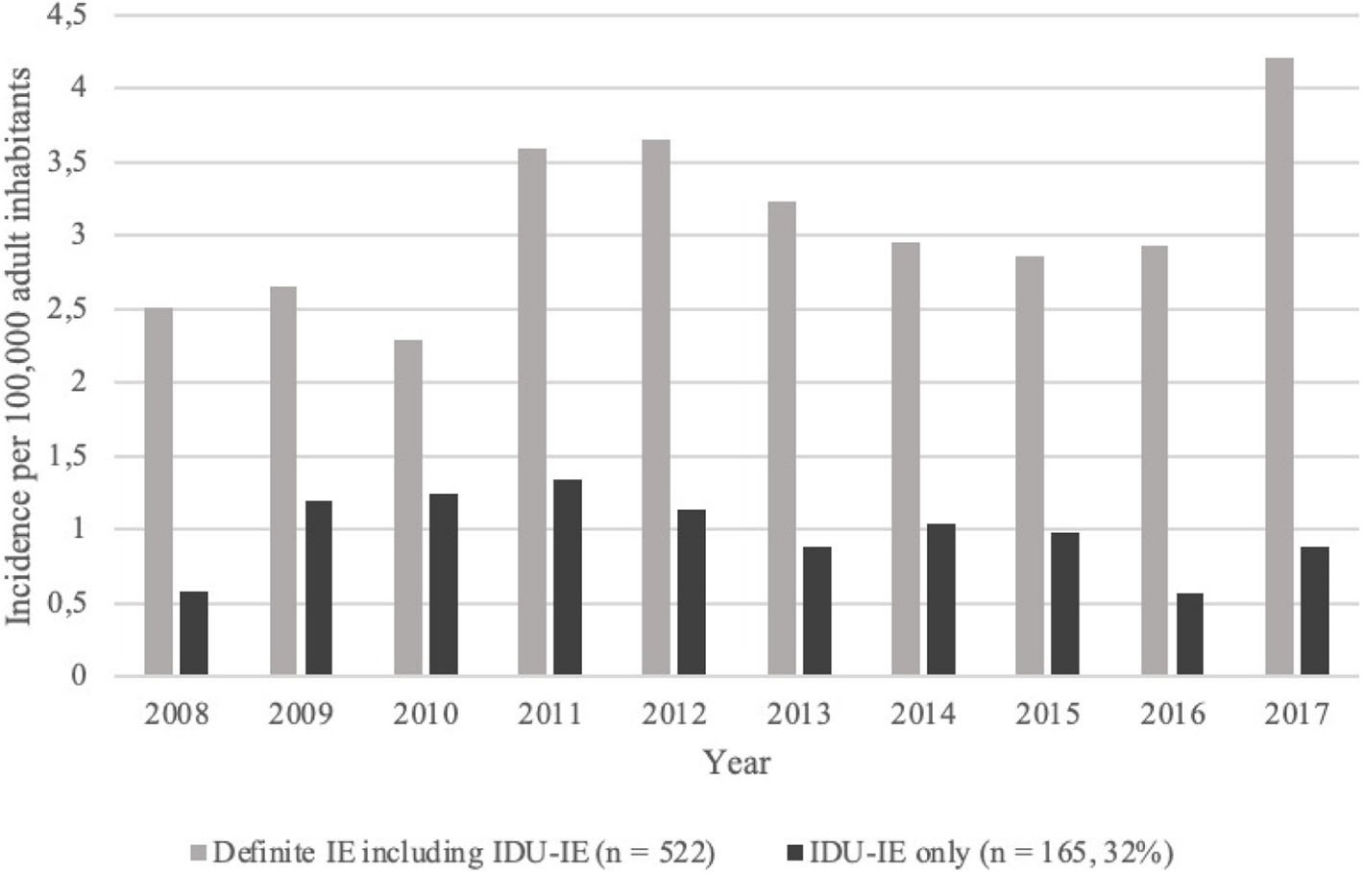
Incidences of definite IE and IDU-IE admitted between 2008 and 2017. Incidences are presented in number of cases per 100,000 adult inhabitants in Stockholm county each year. Abbreviations: IDU, injection drug use; IE, infective endocarditis

In total, 522 patients were registered with definite IE at the Karolinska University Hospital between 2008 and 2017, of these 165 (32%) had history of IDU. The incidence of definite IE registered at the Karolinska University Hospital increased during the study period from 2.52 (2008) to 4.21 cases (2017) per 100,000 adult (18 years and older) inhabitants in Stockholm county (*P* <  0.01). Contrasting, there was an overall decrease in incidence of IDU-IE registered at the hospital between 2008 and 2017 (*P* <  0.01), however, an initial increase between 2008 and 2011 was seen from 0.58 to 1.34 cases per 100,000 adult inhabitants (*P* <  0.01, Fig. 1), but from 2012 to 2017, there was a decrease in incidence from 1.14 to 0.89 cases per 100,000 adult inhabitants in Stockholm county (P <  0.01, Fig. 1). Further, the rate of patients with IDU-IE were lower during the winter compared to the other seasons (*P* = 0.01).

The clinical characteristics of the patients with IDU-IE and non-IDU-IE are presented in Table 1. About two thirds of the included patients with IDU-IE were male (110 patients, 67%), which was the same percentage as among non-IDU-IE patients. Mean age among the patients with IDU-IE was 41.6 years (SD 11.9 years), which was mean 22.7 years younger than patients with non-IDU-IE (mean age 64.3 years, SD 16.4 years; *P* <  0.01). A majority of the patients with IDU-IE (101 patients, 61%) presented with vascular phenomena, which was more common than among patients with non-IDU-IE (Table 1). More specifically, spondylitis and pulmonary septic emboli (both presented as symptomatic complications) were more common among IDU-IE compared with patients with non-IDU-IE (spondylitis *n* = 25, 15% vs *n* = 30, 9%; *P* = 0.02, pulmonary septic emboli *n* = 55, 33% vs *n* = 11, 3%; *P* <  0.01). Further, history of previous IE was more common among the patients with IDU-IE, while prosthetic heart valves, known valvular disease, cardiovascular implantable electronic device (CIED)-associated IE were more common among patients with non-IDU-IE (Table 1). During the 6-month follow up, 3 (2%) of the patients with IDU-IE had reported recurrence of IE (i.e. relapse or re-infection), not significantly different from the non-IDU-IE group (*n* = 5, 1%; *P* = 0.72). Of the patients with recurrent IE in the IDU-IE group, one had relapse (i.e. subsequent episode caused by the same microorganism) and the other two were re-infected (i.e. subsequent episode caused by different microorganisms). Of the patients with recurrent IE in the non-IDU-IE group, four had relapse and one was re-infected.

**Table 1:** Characteristics of the patients with IDU-IE and non-IDU-IE admitted between 2008 and 2017

Clinical characteristics	IDU-IE (***n*** = 165 (%))	Non-IDU IE (***n*** = 357 (%))	***P*** value
Women	55 (33)	119 (33)	1.00
Men	110 (67)	238 (67)	1.00
Age, mean (± SD); median (25th and 75th percentiles)	41.6 (11.9); 44 (31, 50)	64.3 (16.4); 67 (56, 76)	**< 0.01**
**Predisposing factors**			
Bicuspid aortic valve	0 (0)	20 (6)	**< 0.01**
Prosthetic valve	12 (7)	83 (23)	**< 0.01**
CIED	2 (1)	38 (11)	**< 0.01**
Rheumatic heart disease	0 (0)	2 (1)	1.00
Congenital heart disease	0 (0)	8 (2)	0.06
History of IE	49 (30)	34 (10)	**< 0.01**
Known valvular disease	3 (2)	78 (22)	**< 0.01**
Heart failure before or under IE treatment	19 (12)	45 (13)	0.72
**Patients fulfilling Duke’s major criteria**			
Blood culture positive for IE	144 (87)	304 (85)	0.78
Echocardiography positive for IE^a^	165 (100)	287 (80)	**< 0.01**
**Patients fulfilling Duke’s minor criteria**			
Fever	150 (91)	304 (85)	0.07
Vascular phenomena	101 (61)	120 (34)	**< 0.01**
Spondylitis	25 (15)	30 (9)	**0.02**
Pulmonary septic emboli	55 (33)	11 (3)	**< 0.01**
New heart murmur	20 (12)	63 (18)	0.11
Immunological phenomenon	2 (1)	19 (5)	**0.03**
**Outcomes**			
Surgery	27 (16)	121 (34)	**< 0.01**
In-hospital mortality	7 (4)	27 (8)	0.15
Relapse during follow up^b^	1 (0)	4 (1)	1.00
Re-infected during follow up^b^	2 (1)	1 (0)	0.24

^a^In total, 101 (61%) of the IDU-IE patients underwent transthoracic echocardiography and 137 (83%) patients underwent transoesophageal echocardiography during hospital stay. ^b^Follow up within 6 months from discharge. Significant *P* values are marked in bold font. Abbreviations: *CIED* cardiovascular implantable electronic device; *IDU* injection drug use; *IE* infective endocarditis; *n* number of patients; *SD* standard deviation

Of the included patients with IDU-IE, 155 (94%) had positive blood cultures. One of the patients that had negative blood culture had positive polymerase chain reaction (PCR) from heart valve surgery (methicillin susceptible *Staphylococcus aureus* - MSSA) (Table 2 and Table 3). Aetiology of *S. aureus* was significantly more common among patients with IDU-IE compared with patients with non-IDU-IE (IDU-IE: *n* = 123, 75%; non-IDU-IE: *n* = 118, 33%; *P* <  0.01). Of the 522 patients, 274 (52%) underwent both TTE and TEE (IDU-IE: *n* = 79, 48%; non-IDU-IE: *n* = 195, 55%), 168 (32%) underwent TEE only (IDU-IE: *n* = 58, 35%; non-IDU-IE: *n* = 110, 31%) and 58 (11%) underwent TTE only (IDU-IE: *n* = 22, 13%; non-IDU-IE: *n* = 36, 10%). Of the patients with IDU-IE, 94%, compared with 80% of the patients with non-IDU-IE (P <  0.01), had findings consistent with IE during examination with echocardiography, the most common manifestation was tricuspid valve vegetation (91 patients, 55%), followed by mitral valve vegetation (47 patients, 28%) and aortic valve vegetation (33 patients, 20%) (Table 4). A significantly higher prevalence of tricuspid and pulmonary valve vegetations was seen among the patients with IDU-IE compared with the non-IDU-IE patients who had higher prevalence of aortic- and mitral valve vegetations as well as abscess and CIED-associated IE (Table 4). Multiple valve vegetations were similar among patients with IDU-IE (*n* = 27 (16%)) and non-IDU-IE (*n* = 46 (13%), *P* = 0.29).

**Table 2:** Aetiology obtained from blood- and valve culture among patients with IDU-IE and non-IDU-IE

	IDU-IE	Non-IDU-IE	***P*** value
**Blood culture results**^a^ **(n (%))**	**144 (87)**	**336 (94)**	**< 0.01**
*Staphylococcus aureus* (MSSA)	110 (67)	116 (32)	**< 0.01**
*Enterococcus faecalis*	14 (8)	34 (10)	0.70
Viridans group streptococci	9 (5)	94 (26)	**< 0.01**
*S. aureus* (MRSA)	3 (2)	3 (1)	0.39
*Bacillus cereus*	3 (2)	0 (0)	–
CoNS	2 (1)	22 (6)	**0.01**
Group A streptococci	1 (1)	1 (0)	0.53
Group B streptococci	0 (0	11 (3)	–
Group G streptococci	1 (1)	6 (2)	0.44
*Klebsiella spp*	1 (1)	0 (0)	–
Candida spp.	0 (0)	1 (0)	–
Unspecified funghi	1 (1)	1 (0)	0.53
**Polymicrobial blood cultures**	**11 (7)**	**7 (2)**	**< 0.01**
MSSA and *E. faecalis*	4 (2)	0 (0)	–
*E. faecalis* and unspecified funghi	1 (1)	0 (0)	–
MSSA and aerobic gramnegative stave	1 (1)	0 (0)	–
MSSA and candida	1 (1)	0 (0)	–
MSSA and group G streptococci	1 (1)	0 (0)	–
MSSA and unspecified funghi	1 (1)	0 (0)	–
MSSA and viridans group streptococci	1 (1)	1 (0)	0.53
Viridans group streptococci and CoNS	1 (1)	0 (0)	–
**Blood culture negative**	**8 (5)**	**19 (5)**	0.82
**Bacteria in valve culture from cardiac surgery**	**9 (5)**	**17 (5)**	0.73
*Staphylococcus aureus* (MSSA)	5 (3)	7 (2)	0.53
Unspecified funghi	2 (1)	1 (0)	–
CoNS	1 (1)	2 (1)	1.00
*Enterococcus faecalis*	1 (1)	2 (1)	1.00

^a^ Single bacteria in blood culture. All patients in the IDU-IE group with positive valve culture had positive blood culture. Two of the patients in the non-IDU-IE group with positive valve culture had negative blood cultures. Of the patients with positive valve culture in the IDU-IE group, one had bacteria that differed between blood culture (MSSA) and valve culture (unspecified funghi). Of the patients with positive valve culture in the non-IDU-IE group, one had bacteria that differed between blood culture (MSSA) and valve culture (viridans group streptococci). Abbreviations: *CoNS* coagulase negative staphylococci; *E* enterococcus; *IDU* injection drug use; *IE* infective endocarditis; *MRSA* methicillin resistant *Staphylococcus aureus*; *MSSA* methicillin susceptible *Staphylococcus aureus*; *n* number of patients; *PCR* polymerase chain reaction; *S* staphylococcus; *spp*. species

**Table 3:** Aetiology obtained from valve PCR among patients with IDU-IE and non-IDU-IE

	IDU-IE	Non-IDU-IE	***P*** value
**Bacteria in valve PCR from cardiac surgery (n (%))**	**19 (12)**	**14 (4)**	**< 0.01**
*Staphylococcus aureus* (MSSA)	15 (9)	2 (1)	**< 0.01**
CoNS	2 (1)	1 (0)	0.24
*Enterococcus faecalis*	2 (1)	0 (0)	–
*Viridans group streptococci*	0 (0)	6 (2)	–

One patient in the IDU-IE group and three patients in the non-IDU-IE group had positive valve PCR (IDU-IE: MSSA; non-IDU-IE: viridans group streptococci, unspecified gram positive bacteria and *Streptococcus bovis*) and negative blood culture, all other patients with positive valve PCR had positive blood cultures. Among the patients with positive valve PCR had positive blood cultures, the bacteria found in blood culture were the same as those found in valve PCR. Abbreviations: *CoNS* coagulase negative staphylococci; *IDU* injection drug use; *IE* infective endocarditis; *MSSA* methicillin susceptible *Staphylococcus aureus*; *n* number of patients; *PCR* polymerase chain reaction

**Table 4:** Manifestations detected by echocardiography among patients with IDU-IE and non-IDU-IE

All manifestations	IDU-IE (***n*** = 165 (100%))	Non-IDU-IE (***n*** = 287 (80%))	***P*** value
Aortic valve vegetation	33 (20)	171 (48)	**< 0.01**
Mitral valve vegetation	47 (28)	162 (45)	**< 0.01**
Tricuspid valve vegetation	91 (55)	23 (6)	**< 0.01**
Pulmonary valve vegetation	7 (4)	2 (1)	**< 0.01**
CIED-associated IE	1 (1)	25 (7)	**< 0.01**
Abscess	4 (2)	27 (8)	**0.03**
**Multiple valve vegetations**			
Aortic and mitral valve vegetation	11 (7)	31 (9)	0.43
Aortic and tricuspid valve vegetation	5 (3)	3 (1)	
Aortic and pulmonary valve vegetation	1 (1)	0 (0)	
Mitral and tricuspid valve vegetation	5 (3)	5 (1)	
Mitral and pulmonary valve vegetation	1 (1)	0 (0)	
Tricuspid and pulmonary valve vegetation	4 (2)	1 (0)	
CIED and tricuspid valve vegetation	0 (0)	6 (2)	

Among the patients listed with, for instance, aortic valve vegetation, some patients had vegetations also on other valves and thus were listed also under the topic “multiple valve vegetations”. Significant *P* values are marked in bold font. Abbreviations: *CIED* cardiovascular implantable electronic device; *IDU* injection drug use, *IE* infective endocarditis

Significantly less of the IDU-IE patients was treated with surgery; 27 (16%), compared with 121 (34%) among the non-IDU-IE patients (*P* <  0.01). The rate of patients with IDU-IE treated with surgery did not change significantly during the study period (*P* = 0.06). The indications for surgery were assessed from the European guidelines for the management of IE [15]. The most common indications for surgery in the IDU-IE-group were heart failure (*n* = 19, 12%), followed by vegetation (*n* = 14, 8%) while vegetation (*n* = 56, 16%) followed by heart failure (*n* = 42, 12%) were the most common indications for surgery in the non-IDU-IE-group.

The most common single valve surgery performed in the IDU-IE group were biological aortic valve replacement (*n* = 6) followed by biological tricuspid valve replacement (n = 5), and one removal of tricuspid valve vegetation. Eight surgical procedures included multiple valve replacement of which 5 cases included tricuspid valve replacement. Among the patients with IDU-IE treated with surgery, none were re-operated during hospital stay nor during follow-up (up to 6 months). One patient with IDU-IE caused by *E faecalis,* that was not treated with surgery during hospital stay was operated during follow-up due to prosthetic (mechanical) mitral valve dehiscence, that was inserted 14 years prior to the IE diagnosis. Among the patients with IDU-IE treated with surgery, one died during hospital stay but none died during follow-up after discharge from hospital. In-hospital mortality was similar among the patients treated with surgery and the patients not treated with surgery, both in the IDU-IE group (1 (4%) of the 27 patients treated with surgery died during hospital stay and 6 (5%) of the 132 patients not treated with surgery died during hospital stay, *P* = 1.00) and in the non-IDU-IE group (12 (10%) of the 121 patients treated with surgery died during hospital stay and 15 (6%) of the 236 patients not treated with surgery died during hospital stay, *P* = 0.23).

In-hospital mortality was similar among patients with IDU-IE (*n* = 7 (4%)), and those with non-IDU-IE (non-IDU-IE *n* = 27 (8%), *P* = 0.15). Among the patients with IDU-IE, the most common cause of death was heart failure (*n* = 4) followed by cardiac arrest (*n* = 1), intracerebral haemorrhage (n = 1) and liver failure (n = 1). Also among patients with non-IDU-IE, the most common cause of death was heart failure (*n* = 15). Among patients with *S. aureus* aetiology, in-hospital mortality was higher among patients with non-IDU-IE compared with the patients with IDU-IE (*n* = 18 (15%) and *n* = 5 (4%), respectively, *P* = 0.01). In the IDU-IE group, 80 (70%) of the patients with *S. aureus* aetiology had right-sided IE, and 35 (30%) had left sided IE. In the non-IDU-IE group, 20 (18%) of the patients with *S. aureus* aetiology had right-sided IE, and 95 (83%) had left sided IE. Hence, of the patients with *S. aureus* aetiology, right sided IE was more common in the IDU-IE group (OR 10.74; *P* <  0.01). In the IDU-IE group, 3 (4%) of the patients with *S. aureus* aetiology and right-sided IE died during hospital stay, which was similar to the in-hospital mortality among the left-sided IE (*n* = 2 (6%); OR 0.64; *P* = 0.64). In the non-IDU-IE group, 2 (10%) of the patients with *S. aureus* aetiology and right-sided IE died during hospital stay, which was similar to the in-hospital mortality among the left-sided IE (*n* = 14 (15%); OR 0.63; *P* = 0.73).

The duration of hospital stay was similar among patients with non-IDU-IE (median 32 days, interquartile range (IQR) 20 days) compared with patients with IDU-IE (median 32 days, IQR 14 days; *P* = 1.00). The duration of antibiotic treatment was longer in the non-IDU-IE group (median 30 days, IQR 10 days) compared with the IDU-IE group (median 28 days, IQR 12 days; *P* = 0.02). Patients with left-sided IDU-IE caused by *S. aureus* had similar antibiotic treatment durations (median 28 days, IQR 12 days) as patients with *S. aureus* and right-sided IDU-IE (median 28 days, IQR 9 days; P 1.00). The patients that died during hospital stay had shorter duration of antibiotic treatment (median 23 days, IQR 13 days) compared with the patients that survived (median 30 days, IQR 11 days; *P* <  0.01).

## Discussion

This study presents history of previous IE was more common in the IDU-IE group, however a lower extent of the patients with IDU-IE had prosthetic heart valves and known valvular disease compared with those with non-IDU-IE. The rate of patients with positive echocardiography was higher among patients with IDU-IE compared with non-IDU-IE, which could possibly be explained by the higher rate of right-sided valve vegetations in the IDU-IE group, which can be easier to identify also with TTE. Another reason could be the younger age and less comorbidities in the IDU-IE group compared with the non-IDU-IE group, which could have contributed to more frequent use of TEE in this group, which has higher sensitivity and specificity for IE, compared with TTE.

The patients with IDU-IE were younger than the patients with non-IDU-IE. Supportive to our results, previous studies of IDU-IE present younger populations compared to patients with non-IDU-IE [2, 3, 8, 17]. The population of patients with IE is known to have a domination of male gender, the ratio of male to female patients are commonly presented as 3:1 or 2:1 [17–19]. However, Wurcel et al., presented a prevalence of female gender of 40.9% among IDU-IE and even higher in the age group of 15–34-year-old patients with IDU-IE (3). The population of IDU-IE patients in our study had a similar rate of female patients in the age group 15–34 years as in that study. This finding suggest that the demographics of patients with IDU-IE are shifting towards a population of younger, female patients compared with previously reported demographics of the IDU-IE population [3, 17].

Previous studies have described an increasing incidence of IE in general, and of IDU-IE specifically [1, 8]. In our study, the incidence of definite IE registered at the Karolinska University Hospital increased during the study period, but the incidence varied among the patients with IDU-IE. There was an increase in incidence of IDU-IE registered at the hospital between 2008 and 2011 but from 2011 to 2017, there was an overall decrease. Further, the rate of patients with IDU-IE were lower during the winter compared to the other seasons, which according to our knowledge, has not been presented before. Seasonal changes in infectious diseases have been described before [20]. Most bacteria tend to increase with rising temperature [20], which could possibly explain the lower rate of IE during the winter season. The decrease in incidence of IDU-IE after 2011 might be explained by a syringe exchange programme that was introduced in Stockholm at that time. Such programs have shown to reduce HIV and hepatitis infections, but also have been suggested to decrease bacterial infections among IDU, such as IE [2].

*S. aureus* was the most common aetiology among the patients with IDU-IE, and significantly more common compared with patients with non-IDU-IE. This finding is supported by previous studies that have shown *S. aureus* was the most common aetiology among IDU-IE [1, 10, 21]. The high prevalence of *S. aureus* among the IDU-IE may explain the higher prevalence of vascular phenomena such as spondylitis and pulmonary septic emboli among the IDU-IE compared with the non-IDU-IE, findings that are supported by Lassen et al. [21]. In our study, patients with *S. aureus* aetiology and non-IDU-IE had higher in-hospital mortality compared with the patients with *S. aureus* aetiology and IDU-IE. Further, left-sided IE was more common among the patients with *S. aureus* aetiology and non-IDU-IE. Previous studies of patients with IE have presented higher in-hospital mortality among patients with *S. aureus* aetiology and left-sided IE, compared with right-sided IE [22, 23]. Further, polymicrobial infection was more common among the patients with IDU-IE compared with the patients with non-IDU-IE. This finding is supported by a study by Sousa et al. [24]. Polymicrobial aetiology together with prior IE, left-sided IE, intracardiac complications, and stroke were presented as risk factors for 6-month mortality among patients with IDU-IE, according to a multinational study of 7616 patients with IE [25].

In this study, patients with IDU-IE were less treated with surgery compared to patients with non-IDU-IE. This is supported by the multinational study that presented that surgery was less frequently performed in patients with IDU-IE [25]. The rate of patients with IDU-IE treated with surgery did not change significantly during the study period. The in-hospital mortality was similar among patients treated with surgery and those not treated with surgery, both in the IDU-IE and the non-IDU-IE group. However, a previous study presented that IDU-IE was associated with a higher hazard of death or reoperation between 90 and 180 days after first surgery, and that reoperation were less common among non-IDU-IE [9]. Such increased risk in the IDU-IE population should be taken into account for the management of these patients.

In our study, the in-hospital mortality was similar among patients with IDU-IE and non-IDU-IE, although the relatively low absolute numbers of patients could explain the absence of a significant difference. However, supportive to our results are two studies from the U.S., one that showed hospital mortality did not differ between IDU-IE and non-IDU-IE patients and one that presented less hospital mortality among IDU-IE [1, 8]. On the contrary, both studies presented a longer duration of hospital stay among IDU-IE which was not the case in this study [1, 8].

History of previous IE among the admitted patients, were more common in the IDU-IE group compared with the patients with non-IDU-IE. This has been described before [1, 8, 10]. Lassen et al., described 29% of the patients with IDU-IE had recurrence during follow-up [21]. Relapse of IDU has been described as a major risk factor of reinfection in patients with IDU-IE. This increases the risks of potential need for surgical treatment, and death [13]. Hence, treatment of addiction is crucial in patients with IDU-IE [13].

### Limitations

This study was based on data from the Swedish National Registry of Infective Endocarditis. Information about some parameters such as surgical criteria were limited. Surgical criteria were only registered for patients that underwent surgery. Hence, it was not possible to make comparisons of mortality, or other outcomes between patients that had indication for surgery but did not undergo surgery and those with indication that underwent surgery. The registry did include information about some comorbidities, although not all comorbidities needed for a quantification of overall comorbidity using the Charlson comorbidity index. As data obtained from the SRIE were anonymized and did not include any personal data, some patients might have been admitted more than once, and thus included in the registry as a new patient. This could possibly have contributed to competitive risks.

## Conclusions

History of previous IE was more common in the IDU-IE group, in which the patients were younger and had less comorbidities compared with the non-IDU-IE group. However, spondylitis and pulmonary septic emboli were more common in the IDU-IE group. *S. aureus* was the most common aetiology among the patients with IDU-IE, and significantly more common compared with patients with non-IDU-IE. Further, polymicrobial infection was more common among the patients with IDU-IE compared with the patients with non-IDU-IE. Patients with IDU-IE were less treated with surgery compared to patients with non-IDU-IE. Conclusively, as patients with IDU-IE tend to have higher recurrence of IE compared with patients with non-IDU-IE and tend to present with severe complications and polymicrobial infections, these factors should be taken into account for the management of these patients.

## Data Availability

The data that support the findings of this study were taken from the SRIE, but restrictions apply to the availability of these data, which were used under license for the current study, and so are not available publicly. However, data are available from the first author upon reasonable request and with permission of the SRIE.
